# Multivessel Coronary Artery Fistula Presenting as Coronary Steal Syndrome Leading to Cardiac Arrest

**DOI:** 10.7759/cureus.8358

**Published:** 2020-05-30

**Authors:** Muhammad F Ahmed, Anaam Mubin, Rumman Syed, Abdullah K Mahmood, Sonu Sahni

**Affiliations:** 1 Internal Medicine, Brookdale Hospital Medical Center, Brooklyn, USA; 2 Internal Medicine, Saba University School of Medicine, The Bottom, NLD; 3 Internal Medicine, Brookdale University Hospital and Medical Center, New York, USA; 4 Internal Medicine, Brookdale University Hospital Medical Center, New York, USA; 5 Research Medicine, New York Institute of Technology College of Osteopathic Medicine, New York, USA; 6 Primary Care, Touro College of Osteopathic Medicine, New York, USA

**Keywords:** coronary artery fistula, coronary cameral fistula, left anterior descending, ventricular fibrillation, sudden cardiac death

## Abstract

The coronary steal phenomenon refers to myocardial ischemia caused by the diversion of blood away from normal myocardial circulation. A coronary artery fistula (CAF) is an abnormality of the coronary anatomy characterized by the aberrant termination of a coronary artery or its branches into cardiac chambers or great vessels. Although CAFs are often thought to be asymptomatic, fistulas that diverge a significant amount of blood flow and decrease the normal perfusion of myocardial tissue can cause ischemia and can present with acute coronary syndrome. We describe a unique case of a 70-year-old woman with no coronary artery disease (CAD) undergoing ventricular fibrillation and sudden cardiac arrest from myocardial ischemia secondary to coronary steal brought on by multivessel CAFs. This case was unique in that fistulas originating from the left anterior descending and from the circumflex artery draining into the left heart chambers are the least frequently observed. To our knowledge, only two other reports in the literature, demonstrating sudden cardiac arrest in patients with left anterior descending to left ventricle fistulas with no CAD, exist. The case presented, along with the literature reviewed, demonstrates that CAFs may be an important part of the differential diagnosis of symptoms of chest pain and myocardial ischemia, particularly in middle-aged adults with no history of coronary artery disease or related comorbidities.

## Introduction

A coronary artery fistula (CAF) is an abnormality of the coronary anatomy characterized by an aberrant termination of a coronary artery or its branches into cardiac chambers or great vessels. Most CAFs are congenital in origin, though uncommonly, they may also be acquired complications of chest trauma or invasive cardiac procedures such as coronary artery bypass grafting or valve replacement [1]. The exact prevalence of CAFs is difficult to determine but they have been reported to be present in 0.002% of the general population and are found in 0.2%-0.25% of individuals undergoing cardiac catheterization [2]. Although more than 50% of cases are thought to be asymptomatic, fistulas that diverge a significant amount of blood flow and decrease the normal perfusion of myocardial tissue can cause ischemia and can present as acute coronary syndrome (ACS) [3]. Only a few cases of myocardial ischemia caused by coronary steal secondary to CAFs in patients with no angiographic evidence of coronary artery disease (CAD) have been reported in the literature. Herein, we describe a unique case of a 70-year-old woman with no CAD who suffered ventricular fibrillation and sudden cardiac arrest from myocardial ischemia secondary to coronary steal brought on by multivessel CAFs.

## Case presentation

**Figure 1:**
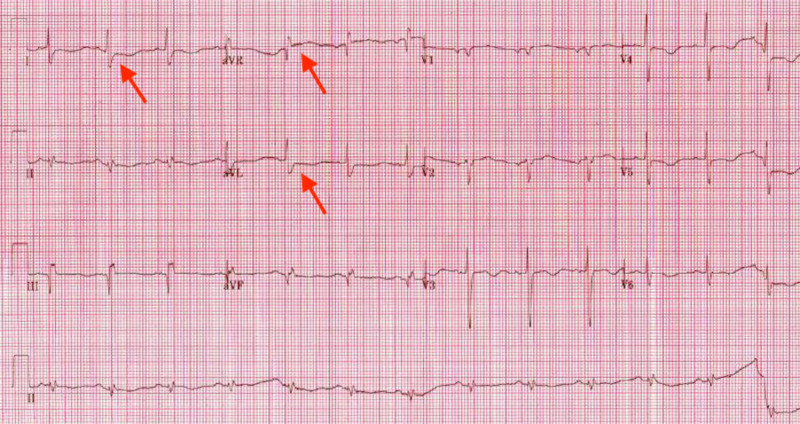
Electrocardiogram revealed ST-segment elevation in lead aVR and ST-segment depression in I, aVL with T wave inversion in multiple leads.

A 70-year-old-female with a past medical history of diabetes mellitus, hypertension, and hyperlipidemia presented with a witnessed cardiac arrest at home. The patient was gardening in her backyard when she suddenly collapsed from a standing position. On arrival of emergency medical services (EMS), she was noted to be pulseless and the rhythm was determined to be ventricular fibrillation. The advanced cardiac life support (ACLS) protocol was initiated by EMS, the patient was shocked seven times, received a total of 7 mg epinephrine, 88 mEq of bicarbonate, 1 gm calcium gluconate, 150 mg amiodarone, and 2 gm magnesium sulfate. The patient was intubated in the field and return of spontaneous circulation was noted after 45 minutes. The rhythm was noted to change from ventricular fibrillation to pulseless electrical activity (PEA), then Torsades de pointes, which reverted to normal sinus rhythm en route to the hospital. On arrival at the hospital, her vitals were noted to be a heart rate of 88 beats per minute, blood pressure of 128/82 mmHg, oxygen saturation of 98%, with respiratory rate of 18 breaths per minute. Her chest was clear to auscultation and first and second heart sounds were heard. The patient was noted to be vomiting and diaphoretic. The electrocardiogram (ECG) on arrival revealed ST-segment elevation in lead aVR and ST-segment depression in I, aVL with T wave inversion in multiple leads, suggestive of possible multivessel coronary artery disease (Figure 1).

**Figure 2:**
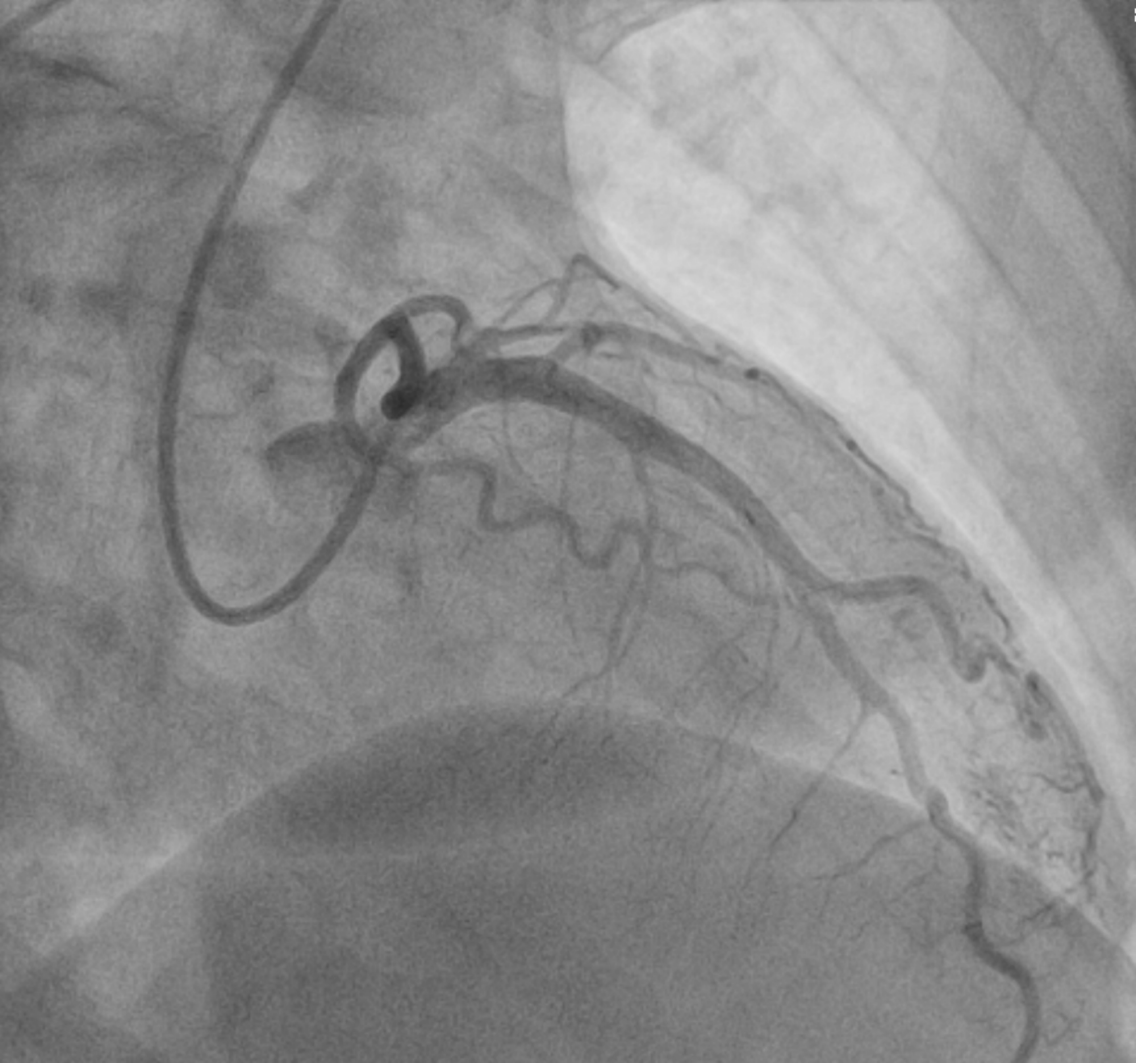
Cardiac catheterization showing no significant coronary artery disease.

**Figure 3:**
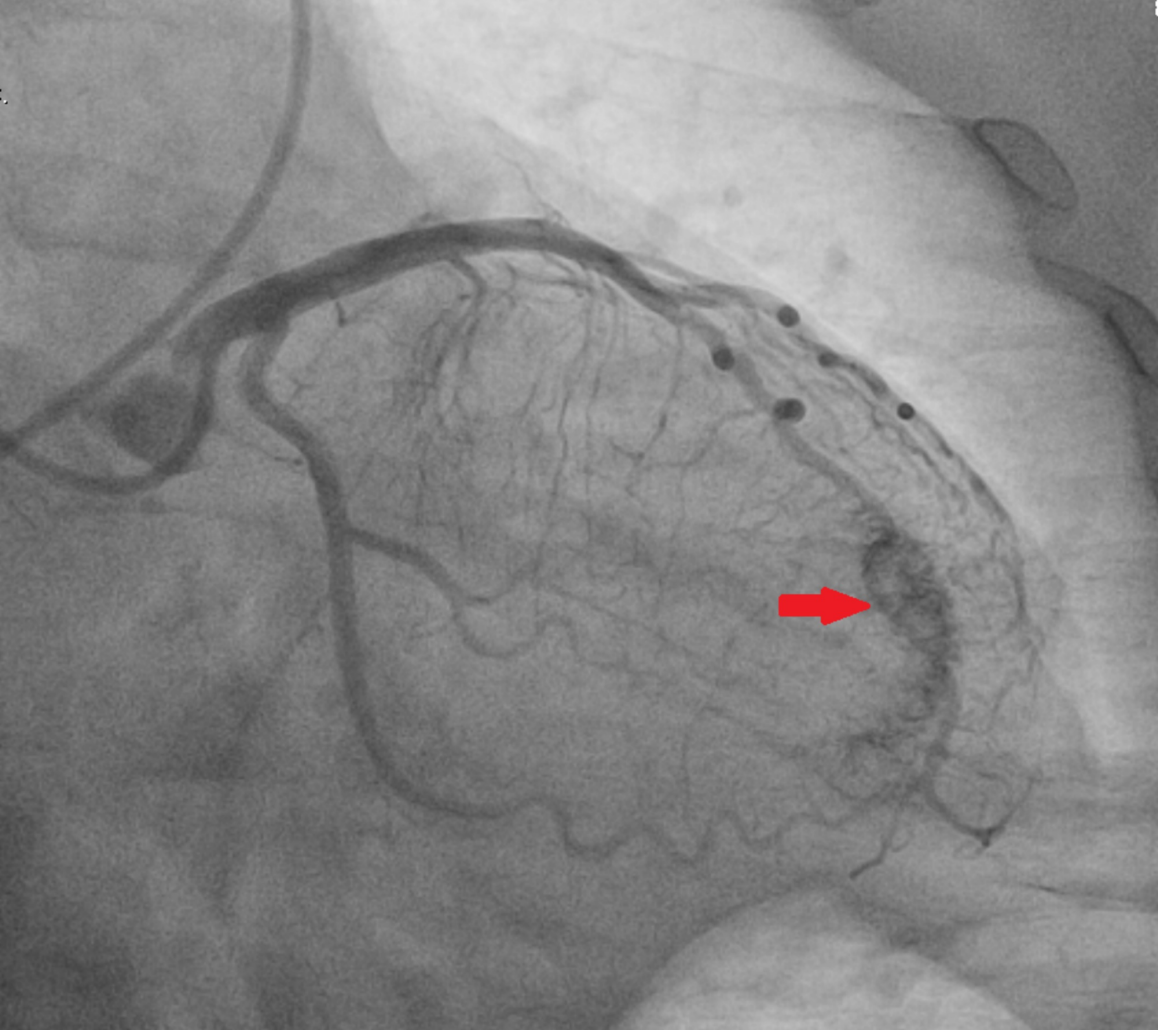
Left anterior descending artery and circumflex artery fistula to the left ventricle was identified (arrow).

Initial labs were significant for glucose 539 mg/dL, creatinine 1.57 mg/dL, alanine transaminase (ALT) 1161 U/L, aspartate transaminase (AST) >1500 U/L, troponin (intact) <0.012 ng/mL, and venous blood gas (VBG) showed pH of 6.87 with partial pressure of carbon dioxide (pCO2) of 72 mmHg. An emergent cardiac catheterization was performed, which revealed no significant CAD (Figure 2). However, the left anterior descending artery (LAD) and circumflex artery fistula (LCx) to the left ventricle (LV) were identified (Figure 3).

Subsequently, the patient had an episode of hematochezia and hypotension that required fluid and vasopressor support. Hematochezia was deemed likely due to ischemic colitis secondary to hypotension and no intervention was performed. The patient had a computed tomography angiogram (CTA) of the chest and pulmonary embolism (PE) was ruled out. During the course of hospitalization, she had no malignant arrhythmia. Implantable cardioverter-defibrillator (ICD) placement was recommended, but the patient requested a transfer to an outside hospital for further management and ICD placement.

## Discussion

The coronary steal phenomenon refers to myocardial ischemia caused by the diversion of blood away from normal myocardial circulation. A fistula communicating with a larger cardiac chamber represents a favorable lower resistance pathway for blood flow relative to the network of smaller diameter coronary vessels. In our patient, a LAD and circumflex artery fistula with the left ventricle was identified. Clinical manifestations may be present depending on the size and point of diversion of the fistula from a main vessel and the subsequent hemodynamic instability caused by the aberrant communication [1]. Most cases of coronary steal syndrome are associated with CAFs in older patients with no CAD, which present as stable angina although unstable angina and arrhythmias have also been rarely observed as in our case [4-8]. This case was also unique in that fistulas originating from the left anterior descending and from the circumflex artery draining into the left heart chambers are the least frequently observed [2]. To our knowledge, only two other reports in the literature exist demonstrating sudden cardiac arrest in patients with LAD to LV fistulas with no CAD [4,7]. A study that reviewed the coronary angiograms of 7267 patients found that of the 24 patients with CAFs, eight had multiple CAFs draining exclusively into the left ventricle with no atherosclerotic coronary artery disease and of these, three had suffered myocardial infarctions [4]. Although rare, these reports, along with this case presented, demonstrate that the coronary steal in cases of CAFs can be significant enough to cause potentially fatal outcomes.

A high degree of clinical suspicion is required, as the diagnosis of CAFs can be challenging. In most cases, CAFs are identified during emergent catheterizations or routine coronary angiography, and these modalities are considered the gold standard for diagnosis [5,9-10]. Contrast-enhanced electron beam tomography with three-dimensional reconstruction has been shown to be a reliable non-invasive technique for identifying the course of anomalous coronary arteries [11]. Transthoracic echocardiography may also be useful in localizing the site of drainage of the fistula, although it is limited to significantly enlarged CAFs with minimal tortuosity [9-10]. Multidetector computed tomography and magnetic resonance imaging are alternative methods of detecting CAFs that can be used adjunctively with angiography to delineate the origin, course, and/or relation to other structures [9-10].

In our case, the patient was given an antiarrhythmic (amiodarone) but no interventions were done, as the patient elected to be transferred to a different institution. Regardless, no official guidelines for the management of CAFs currently exist but transcatheter closure, surgical ligation, or pharmacological management have all been shown to improve outcomes and decrease the recurrence of symptoms. Whether one technique is superior to the other in specific clinical scenarios is not well-known and information on this matter is largely based on low-tier levels of evidence. This is not surprising given the rarity and large degree of variation in anatomy and subsequent clinical manifestations associated with this anomaly. The best approach seems to combine expert judgment based on the specifics of the particular case at hand with shared decision-making with the patient. In cases where there were multiple microfistulae draining into the LV, surgical or percutaneous closure was technically difficult and medical management with beta-blockers proved sufficient to relieve symptoms and prevent recurrence [5,7-8]. Nitrates may not improve and may even precipitate anginal symptoms by increasing flow through the fistulas and worsening coronary steal as reported in two cases [5,12]. Beta-blockers seem to be the medication of choice in patients who present with stable angina, but a closer analysis of specific cases with LAD to LV fistulas with no CAD showed those with unstable angina were refractory to B-blocker therapy [5,7-8,12-13]. In such cases, invasive interventions, such as transcatheter closure or ligation, are more likely to be indicated to prevent future complications. In addition, it is important for clinicians to know that CAFs may be the cause of complications related to prior cardiac interventions. In a case by Malyshev et al., it was noted that a CAF was responsible for multiple defibrillator shocks [14].

## Conclusions

Depending on their effects on myocardial hemodynamics, CAFs can present clinically with a variety of symptoms or be asymptomatic, incidental findings. The case presented, along with the literature reviewed, demonstrates that CAFs may be an important part of the differential diagnosis of symptoms of chest pain and myocardial ischemia, particularly in middle-aged adults with no history of coronary artery disease or related comorbidities.
